# Exploring the mechanism of action of *Phyllanthus emblica* in the treatment of epilepsy based on network pharmacology and molecular docking

**DOI:** 10.1097/MD.0000000000041414

**Published:** 2025-02-14

**Authors:** Longfei Xiao, Wenjun Chen, Wenlong Guo, Hailin Li, Rong Chen, Qinghua Chen

**Affiliations:** a College of Ethnic Medicine, Yunnan University of Chinese Medicine, Kunming, Yunnan, China; b Yunnan Key Laboratory of Dai and Yi Medicines, Yunnan University of Chinese Medicine, Kunming, Yunnan, China.

**Keywords:** epilepsy, molecular docking, network pharmacology, *Phyllanthus emblica*, traditional Chinese medicine

## Abstract

This study explores the mechanism of *Phyllanthus emblica* in treating epilepsy (EP) through network pharmacology and molecular docking. The Traditional Chinese Medicine Systems Pharmacology Database and Analysis Platform identified the chemical composition of *P emblica*, Swiss ADME screened active components, and Swiss Target Prediction predicted targets. EP-related targets were identified using Gene Cards, OMIM, Drug Bank, TTD, and DisGeNET, and Venny 2.1.0 was used to find intersecting targets. Protein–protein interaction network analysis was conducted with STRING and Cytoscape. Chem 3D and Pymol were used for structural optimization and molecular docking was performed with AutoDock Tools 1.5.7 and Vina. Fifty-three active components and 126 intersecting targets were identified. Gene Ontology analysis revealed 3416 biological processes, 287 cellular components, and 457 molecular functions. Kyoto Encyclopedia of Genes and Genomes pathways showed neuroactive ligand–receptor interactions, nitrogen metabolism, and serotonergic synapses as key pathways. Molecular docking indicated strong binding energies between *P emblica* core components and targets, especially 2-ethylhexyl ester with MAPK3, luteolin with SRC, and kaempferol with MAPK1. This study explores the therapeutic potential of *P emblica* in treating EP through network pharmacology and molecular docking. A total of 53 active components were identified, with key compounds like 2-ethylhexyl ester, phyllanthin, luteolin, and kaempferol targeting critical proteins such as SRC, AKT1, APP, MAPK3, and MAPK1. These targets are involved in pathways related to synaptic transmission, oxidative stress, and inflammation, indicating potential neuroprotective and anti-inflammatory effects. Gene Ontology analysis highlighted the regulation of synaptic activity, while Kyoto Encyclopedia of Genes and Genomes pathway analysis emphasized pathways like neuroactive ligand–receptor interactions and serotonergic synapses. Molecular docking demonstrated strong binding affinities between active components and core targets, supporting the effectiveness of *P emblica* in modulating neuronal excitability and reducing neuroinflammation. These findings provide a theoretical basis for its clinical application in EP management.

## 1. Introduction

Epilepsy (EP) is a brain disorder characterized by transient signs or symptoms caused by abnormal excessive or synchronous neuronal activity in the brain.^[1]^ Over 50 million people worldwide are affected by EP, with approximately 125,000 deaths attributed to the disease annually.^[2]^ In China, there are about 9.8 million EP patients,^[3]^ but only about one-third of them receive effective treatment. EP can be secondary to stroke and traumatic brain injury, with approximately 26% of adult patients and 30% to 40% of pediatric patients having intellectual disabilities.^[4]^ The majority of currently used anti-EP drugs can cause dizziness, somnolence, and fatigue, and some specific drugs may even result in severe allergic reactions, liver damage, osteoporosis, and other adverse effects.^[5]^ Compared to synthetic drugs, natural plant medicines tend to have fewer or no side effects in treating diseases.^[6]^ Modern research on natural plant medicines has become a trend.

*Phyllanthus emblica* is the fruit of the *P emblica Linn.* plant in the Euphorbiaceae family, known for its effects of clearing heat and cooling blood, aiding digestion and stomach health, and promoting saliva production to quench thirst. *P emblica* is a commonly used medicinal material in Yi medicine, recorded in Yi medical classics such as “Qi Su Shu” and “Xian Yao Jing,” and is used to treat diseases such as EP, urinary retention, drunkenness, rheumatism, frostbite, centipede bites, and syphilis.^[7]^ There are many modern pharmacological studies on the treatment of EP with *P emblica*. For instance, Mahaveer Golechha and colleagues.^[8]^ used aqueous-alcoholic extracts of *P emblica* at doses of 300, 500, and 700 mg/kg for a 7-day pretreatment in rats and evaluated them in rats with EP induced by pentylenetetrazole (PTZ). They found that the extracts at doses of 500 and 700 mg/kg could eliminate generalized tonic-clonic seizures of EP, while the 300 mg/kg dose could improve related cognitive deficits. A study^[9]^ induced EP in rats with 10 mg/kg kainic acid, observed behavioral changes, incidence, and the latency of convulsions over 4 hours, and then estimated oxidative stress parameters after sacrificing the rats. It was found that the group pretreated with *P emblica* aqueous-alcoholic extracts at intraperitoneal doses of 500 and 700 mg/kg showed a significant increase in the latency of EP seizures. Furthermore, *P emblica* capable of extending the seizure latency period, with no detrimental impact on the renal and hepatic functions of rats when administered orally over the long term.^[10]^

This study utilized network pharmacology methods to analyze the effective components of *P emblica* fruit, predict its action targets and related signaling pathways, and employ molecular docking techniques to investigate the interactions between core targets and active components. This research explores the mechanism of *P emblica* in treating EP, providing a theoretical basis for its clinical application in the treatment of EP.

## 2. Materials and methods

### 2.1. Construction of the *P emblica–*active components and target database

The chemical components of *P emblica* were obtained through the Traditional Chinese Medicine Systems Pharmacology Database and Analysis Platform (https://www.91tcmsp.com/)^[11]^ and supplemented with chemical components from literature.^[6]^ The sdf files of *P emblica* compounds were acquired from the PubChem database (https://pubchem.ncbi.nlm.nih.gov)^[12]^ and imported into the SwissADME database (http://www.swissadme.ch/faq.php).^[13]^ The selection criteria included a high gastrointestinal absorption rate (GI absorption) labeled as “High” and drug-likeness with 2 or more “Yes” ratings.^[14]^ The obtained effective active components of *P emblica* were then imported into the Swiss Target Prediction database (http://swisstargetprediction.ch),^[15]^ with a confidence level of ≥ 0.1 as the selection standard to acquire related targets.

### 2.2. Screening of EP disease targets

Disease targets for EP were identified using the Gene Cards (https://www.genecards.org), OMIM (https://www.omim.org),^[16]^ Drug Bank (https://go.drugbank.com),^[17]^ TTD (https://db.idrblab.net),^[18]^ and DisGeNET (https://www.disgenet.org)^[19]^ databases with “epilepsy” as the search term. The UniProt (https://www.uniprot.org)^[20]^ was then accessed to select “reviewed” and “human species” standardized protein targets. The disease targets obtained from the 5 databases were merged and deduplicated to obtain the EP disease targets.

### 2.3. Construction of the *P emblica* components–EP core target protein–protein interaction (PPI) network

To clarify the interactions between the targets related to the chemical components of *P emblica* and the targets of EP, the intersection of the 2 sets of targets was obtained, and a Venn diagram was drawn using the online tool Venny 2.1.0 (https://bioinfogp.cnb.csic.es/tools/venny/index.html).^[21]^ The intersection targets were then entered into the STRING 12.0 database (https://string-db.org)^[22]^ to construct a PPI network. The species was set to “Homo sapiens,” and the minimum required interaction value was set to “highest confidence” (>0.7). Isolated nodes were removed, and the file was saved in tsv format and imported into the Cytoscape 3.9.1 software for visualization analysis. The Network Analyzer plugin was used to obtain the topological characteristic attribute values of the network, and core targets with a degree of ≥ 18 were selected.

### 2.4. Functional prediction of *P emblica–*EP intersection targets

The intersection targets of *P emblica* and EP were imported into the Microbioinformatics platform (http://www.bioinformatics.com.cn) for Gene Ontology (GO) and Kyoto Encyclopedia of Genes and Genomes (KEGG) enrichment analysis.^[23]^ The biological processes, cellular components, molecular functions, and KEGG pathway enrichment analysis involved in the treatment of EP by *P emblica* were obtained. Based on the drug–disease intersection targets and the main signaling pathways–target points obtained from the KEGG pathway enrichment analysis, the *P emblica*–active components–targets–pathways network was constructed. The Microbioinformatics platform was used to draw the GO_KEGG enrichment analysis diagram and the mapping diagram of the signaling pathway targets ranked first by *P*-value, and the results were analyzed.

### 2.5. Molecular docking of key active components of *P emblica* with EP core targets

The core targets of *P emblica*–EP were entered into the UniProt (https://www.uniprot.org)^[24]^ to obtain the UniProt IDs of the targets. The three-dimensional structures of the aforementioned target proteins were obtained from the PDB (RCSB Protein Data Bank) database (https://www.rcsb.org).^[25]^ The molecular structures of the key active components of *P emblica* were optimized using Chem 3D 22.0 software to obtain mol format files. The three-dimensional protein structures were imported into Pymol 2.6 software to remove water molecules and small-molecule compound ligands. Molecular docking was performed using AutoDock Tools 1.5.7 and Vina software to calculate the binding energy. The result files and receptor files were imported into Pymol software for visualization analysis.

## 3. Results

### 3.1. Screening results of *P emblica–*EP intersection targets

**Figure 1.**
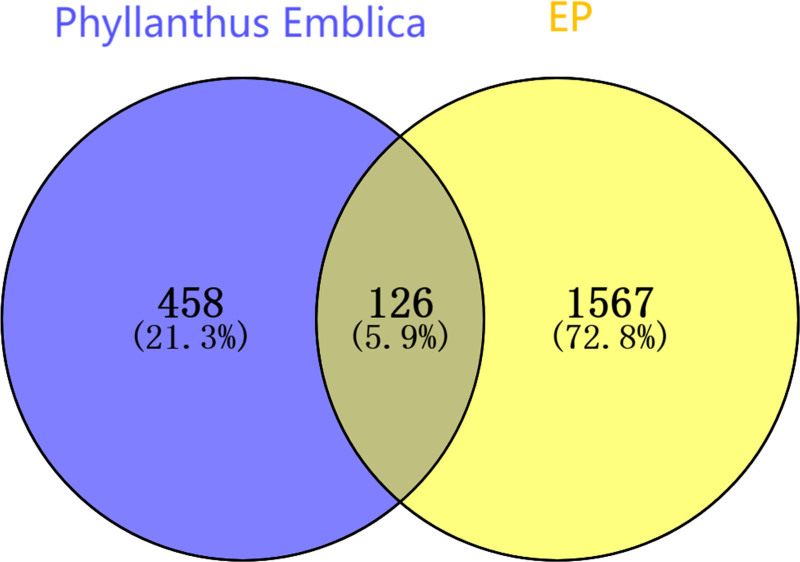
Venn diagram of *Phyllanthus emblica*–EP targets. EP = epilepsy.

A total of 53 effective components of *P emblica* were obtained, with chemical components from the Traditional Chinese Medicine Systems Pharmacology Database and Analysis Platform listed in Table 1 and chemical components supplemented by literature listed in Table 2. A total of 584 predicted target points were identified. Using the Gene Cards database, EP disease targets were screened with a relevance score of ≥ 3, yielding 1114 targets. The OMIM database yielded 666 EP disease targets, the Drug Bank database yielded 68 EP disease targets, the TTD database yielded 54 EP disease targets, and the DisGeNET database yielded 154 EP disease targets with a score >0.1 (Score_gda > 0.1). Raw data of disease targets from different databases, detailed results are shown in Tables S1–S5, Supplemental Digital Content, http://links.lww.com/MD/O364. After merging and deduplicating the obtained disease targets, a total of 1693 targets were acquired. The intersection with the effective component target points of *P emblica* yielded 126 drug–disease target points (Fig. 1).

**Table 1:** Active components obtained from TCMSP.

ID	Active components	PubChem CID	ID	Active components	PubChem CID
YGZ1	Ellagic acid	5281855	YGZ10	Brevifolin	66654
YGZ2	Myristic acid	11005	YGZ11	3,4,5-Trihydroxybenzoic acid	370
YGZ3	NON	2969	YGZ12	Luteolin	5280445
YGZ4	Methylgallate	7428	YGZ13	OHP	11970
YGZ5	Progallin A	13250	YGZ14	3-Ethoxy-4,5-dihy-droxy-benzoic acid	86047602
YGZ6	Pentagalloylglucose	65238	YGZ15	Phyllanthin	358901
YGZ7	Hexanoic acid	8892	YGZ16	Palmitic acid	985
YGZ8	Caprylic acid	379	YGZ17	Quercetin	5280343
YGZ9	Kaempferol	5280863			

TCMSP = The Traditional Chinese Medicine Systems Pharmacology Database and Analysis Platform.

**Table 2:** Active components supplemented by literature.

ID	Active components	PubChem CID	ID	Active components	PubChem CID
YGZ18	Isopropy-l,2-methyl butyrate	522214	YGZ36	Phydroxybenzoic	135
YGZ19	Acetophenone	7410	YGZ37	Vanillic	8468
YGZ20	Octyl-B-D-glucopyranoside	62852	YGZ38	Syringic	10742
YGZ21	3-Phenylphenol	11381	YGZ39	Ferulic	445858
YGZ22	1,2,3-Benzenetriol	1057	YGZ40	L-Ornithine	6262
YGZ23	Heptanoic acid	519189	YGZ41	beta-Hydroxyisovaleric acid	69362
YGZ24	Dimethyl adipate	12329	YGZ42	5-Hydroxytryptamine	5202
YGZ25	Methyl adipate	12328	YGZ43	2-(3,4-Dimethoxyphenyl) ethanamine	8421
YGZ26	Protocatechuic acid	72	YGZ44	Indoleacrylic acid	5375048
YGZ27	P-Coumaric acid	637542	YGZ45	Sinapinic acid	637775
YGZ28	Fisetin	5281614	YGZ46	Palmitic acid methyl ester	8181
YGZ29	Ethyl gallate	3250	YGZ47	Stearamide	31292
YGZ30	5-Hydroxyisophthalic acid	69257	YGZ48	Leucine	6106
YGZ31	3,5-Dihydroxybenzoic acid	7424	YGZ49	Niranthin	13989915
YGZ32	10-Gingerdione	5317591	YGZ50	Caffeic acid	689043
YGZ33	6-Methylgingediacetate	53145002	YGZ51	Chrysin	5281607
YGZ34	7alpha-Hydroxycholesterol	107722	YGZ52	Coumaric acid	637540
YGZ35	2′-Hydroxycinnamaldehyde	5318169	YGZ53	2-ethylhexyl Ester	591313

### 3.2. Screening results of *P emblica–*EP core targets

**Figure 2.**
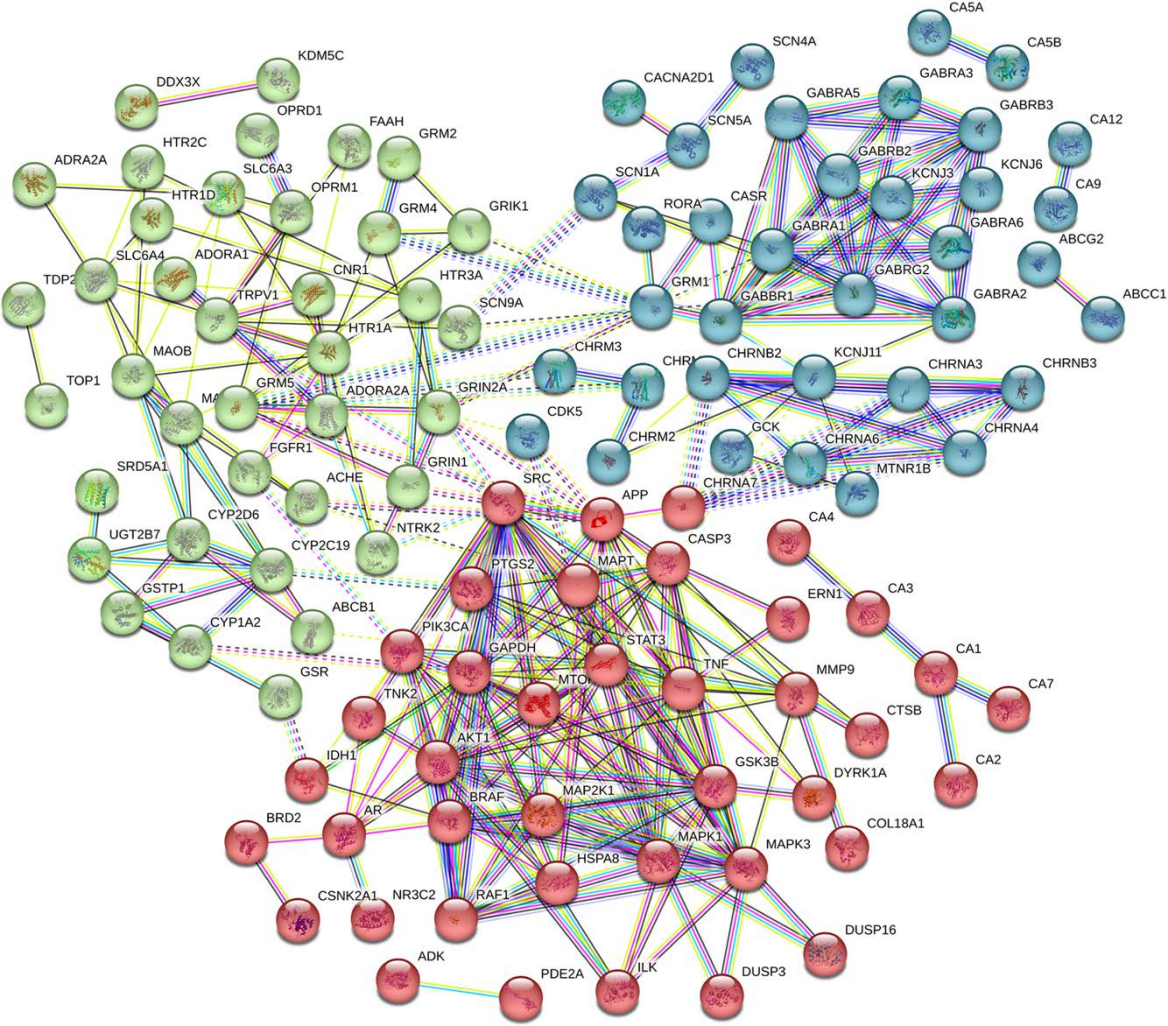
Protein–protein interaction network of intersection targets.

**Figure 3.**
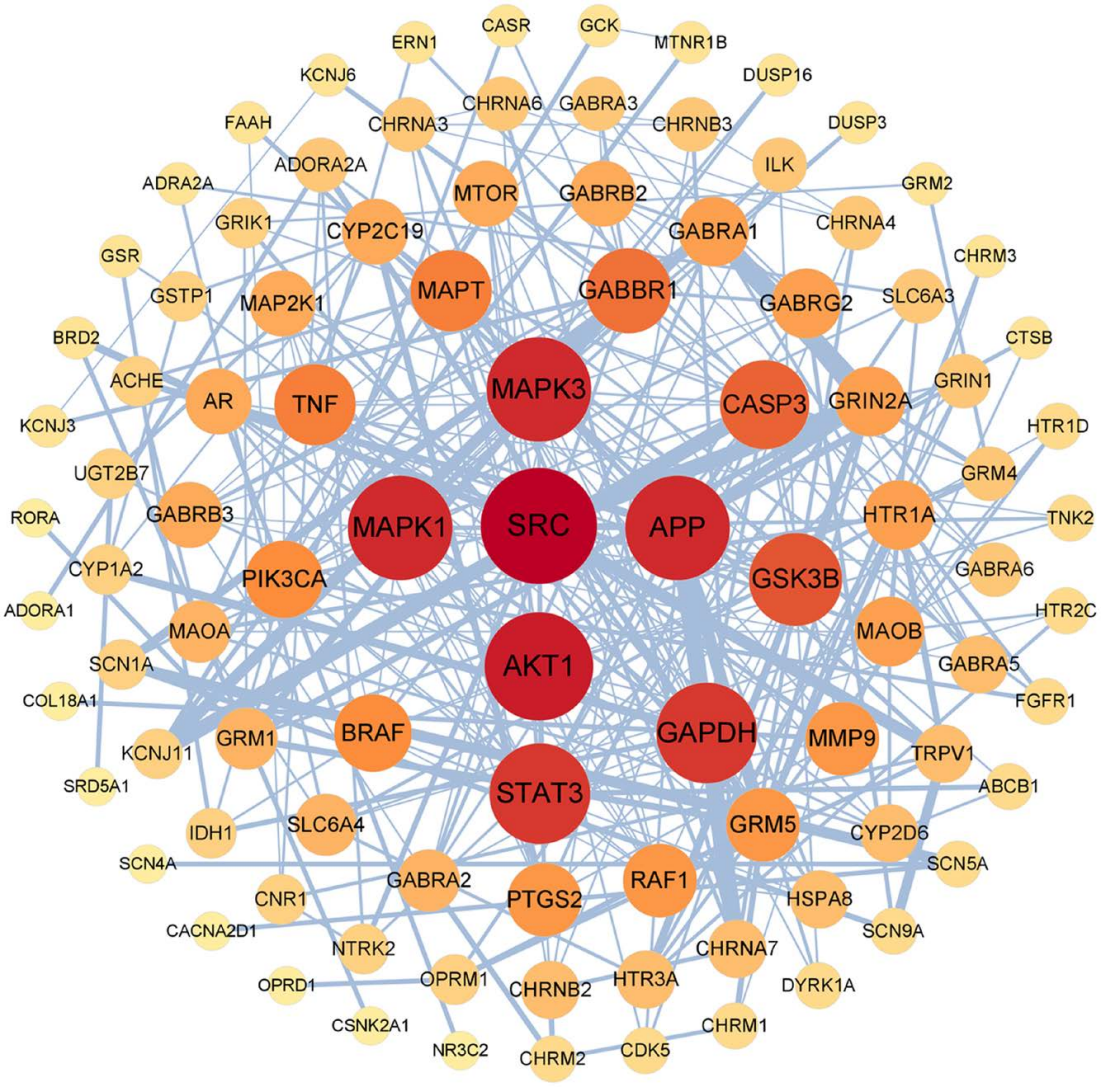
Core targets screening.

A PPI network graph was established based on the 126 intersection targets of *P emblica* and EP (Fig. 2). The intersection targets were imported into Cytoscape 3.9.1 software for visualization analysis (Fig. 3). The core targets of *P emblica*–EP, including SRC, AKT1, APP, MAPK3, and MAPK1, were screened based on a degree value of ≥ 18.

### 3.3. Screening results of active components of *P emblica*

**Figure 4.**
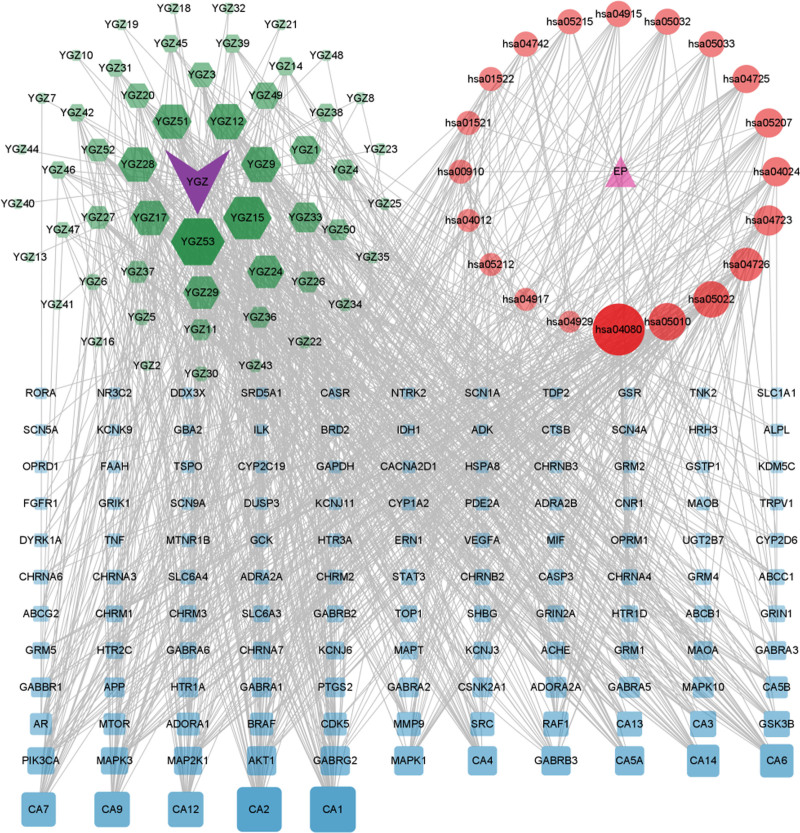
*Phyllanthus emblica*–active components–target–pathway network. *Note*: green: active components; red: pathway; blue: target. YGZ: *Phyllanthus emblica*.

The drug–disease intersection targets, the top 20 signaling pathways based on *P*-value, and their associated targets were imported into Cytoscape 3.9.1 software to construct the *P emblica*–active components–targets–pathways network (Fig. 4). The main active components of *P emblica*, including 2-ethylhexyl ester, phyllanthin, luteolin, and 53 others, act on the 126 intersection targets. 2-Ethylhexyl ester acts on 42 targets, phyllanthin on 37 targets, phyllanthin and kaempferol both on 29 targets, and fisetin, chrysin, and quercetin all on 28 targets.

### 3.4. Enrichment analysis results of *P emblica–*EP intersection targets

**Figure 5.**
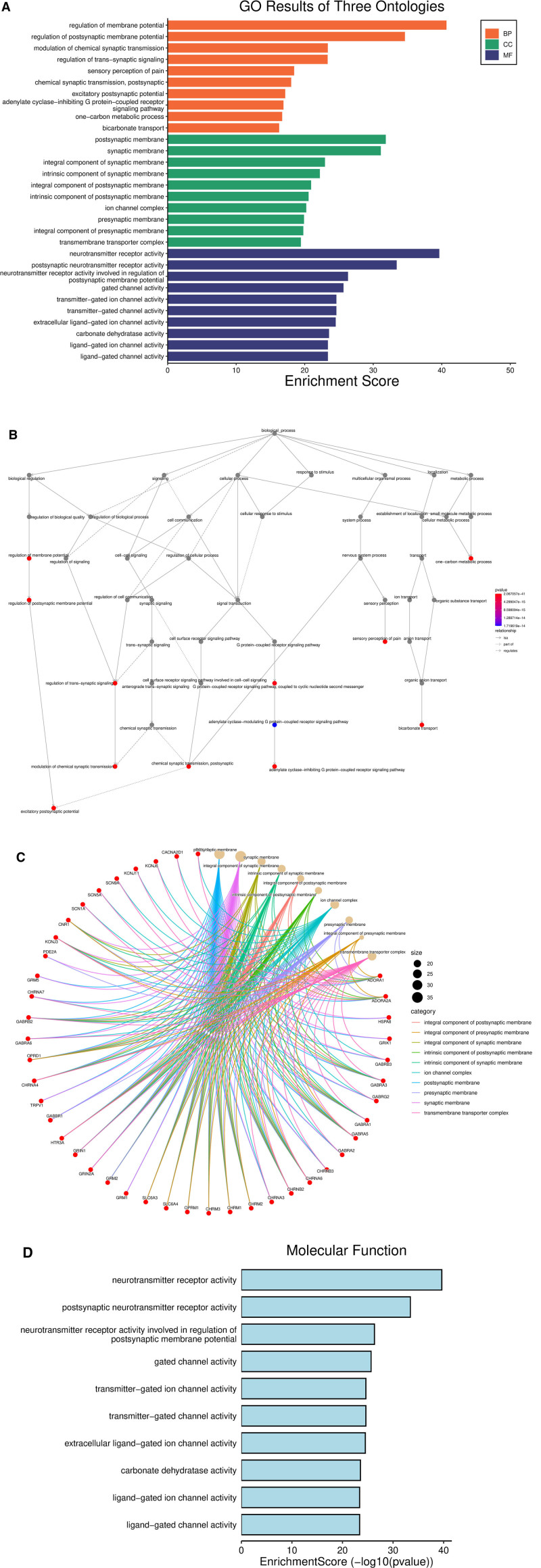
GO enrichment analysis. *Note*: (A) GO results of 3 ontologies; (B) biological processes; (C) cellular components; (D) molecular functions. GO = Gene Ontology.

**Figure 6.**
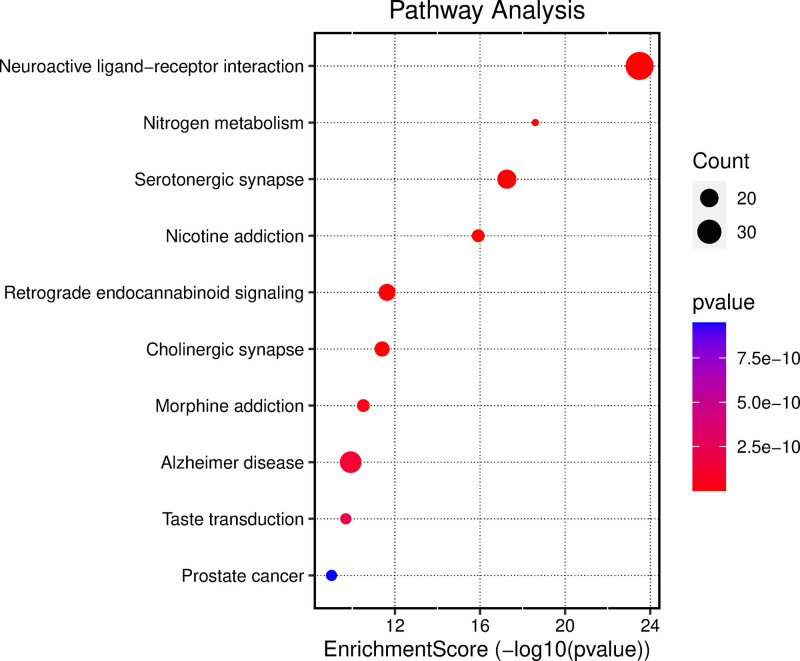
KEGG enrichment analysis. KEGG = Kyoto Encyclopedia of Genes and Genomes.

**Figure 7.**
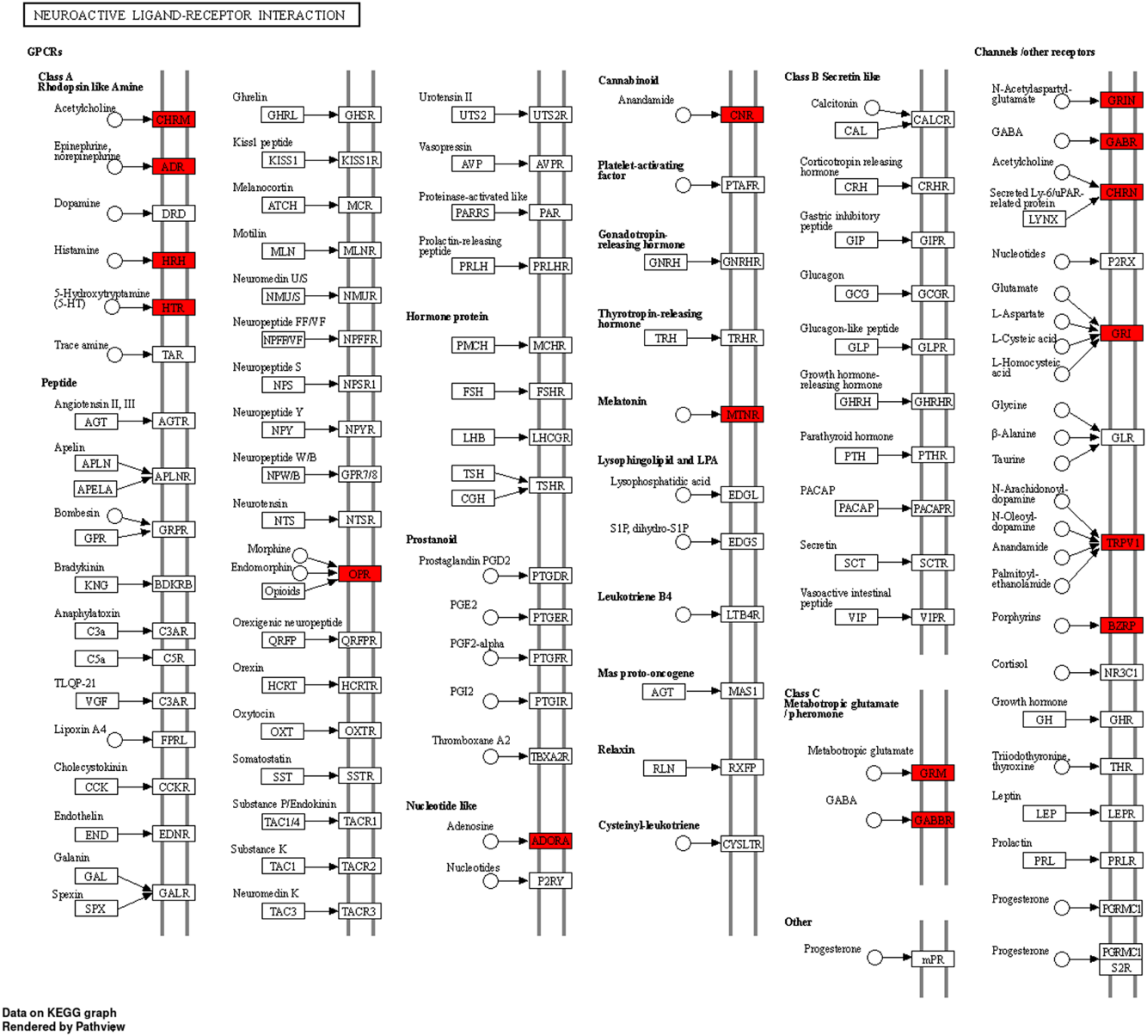
Mapping of neuroactive ligand–receptor interaction targets of the hsa04080 signaling pathway in cytokine–cytokine receptor interactions.

The GO enrichment analysis was based on the top 10 biological processes, cellular components, and molecular functions of the 126 intersection targets of *P emblica*–EP (Fig. 5A). The biological processes mainly include the regulation of chemical synaptic transmission, sensory perception of pain, and adenyl cyclase-activating G protein-coupled receptor activity, among others (Fig. 5B). The cellular components mainly include the postsynaptic membrane, synaptic membrane, and components of the synaptic membrane, among others (Fig. 5C). Molecular functions include neurotransmitter receptor activity, postsynaptic neurotransmitter receptor activity, and neurotransmitter receptor activity involved in the regulation of postsynaptic membrane potential, among others (Fig. 5D). According to the KEGG enrichment analysis, the pathways related to the treatment of EP by *P emblica* include neuroactive ligand–receptor interaction, nitrogen metabolism, and serotonergic synapse, among others (Fig. 6). Among them, the signaling pathway with the highest *P*-value, hsa04080 neuroactive ligand–receptor interaction, is mapped in the context of cytokine–cytokine receptor interaction (Fig. 7). In the figure, red represents the core target-related genes. Request for Permission to Publish Content under the CC-BY license, detailed results are found in File S1, Supplemental Digital Content, http://links.lww.com/MD/O365.

### 3.5. Results of molecular docking of key active components of *P emblica* and *P emblica–*EP core targets

**Figure 8.**
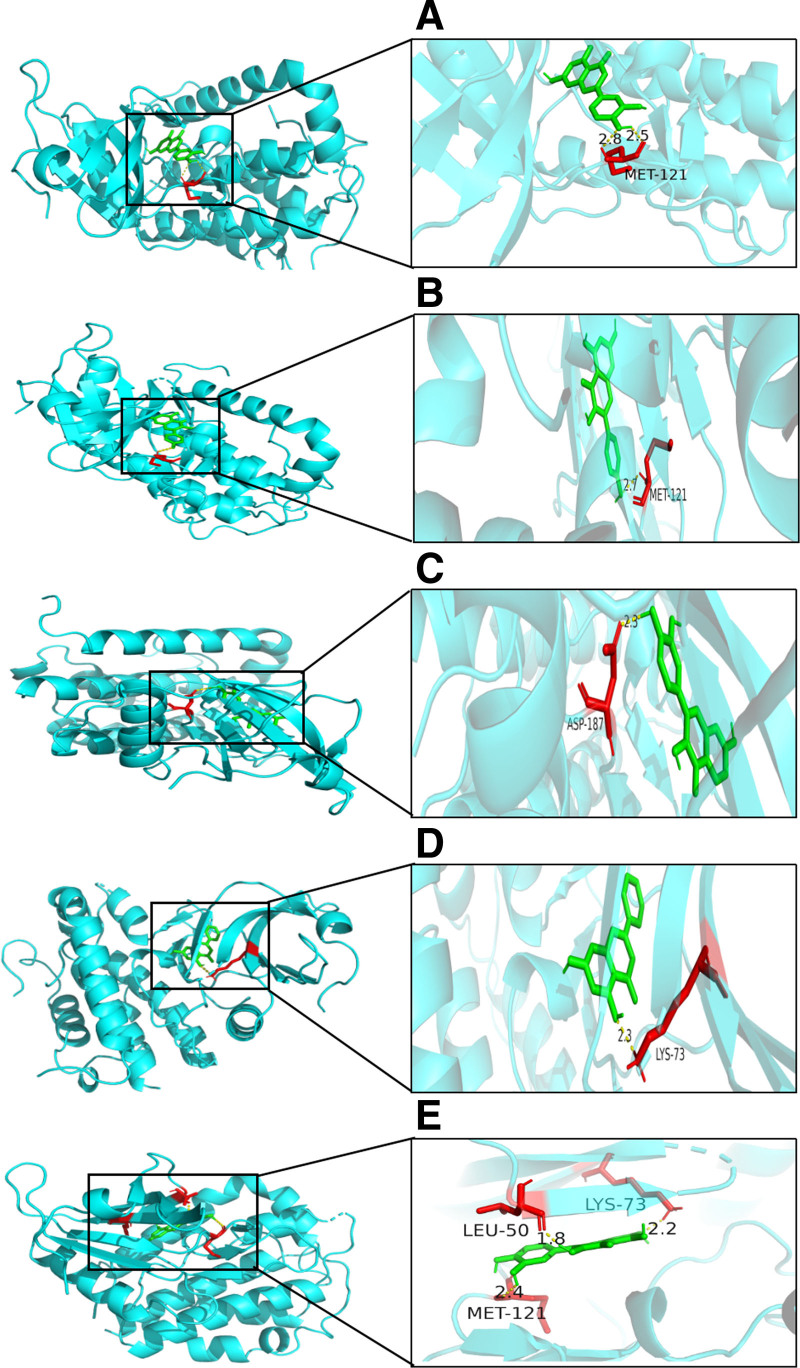
Visualization of molecular docking result. *Note*: (A) luteolin with MAPK3; (B) kaempferol with MAPK3; (C) fisetin with MAPK3; (D) chrysin with MAPK3; (E) quercetin with MAPK3.

The UniProt IDs obtained from the UniProt database, and the PDB IDs for the three-dimensional structures of the target proteins obtained from the PDB database, are presented in Table 3. Molecular docking was performed using the core targets of the drug–disease SRC, AKT1, APP, MAPK3, and MAPK1 as receptors, and key active components of *P emblica*, including 2-ethylhexyl ester, phyllanthin, luteolin, kaempferol, fisetin, chrysin, and quercetin, as ligands. The binding energies of the key active components of *P emblica* with the *P emblica*–EP core targets are presented in Table 4. A molecular binding energy >4.25 indicates the existence of binding capability between the molecule and the target protein; >5.0 indicates superior binding activity; and >7.0 indicates extremely strong binding activity.^[26]^ According to the results, the binding energies of the *P emblica*–EP core targets with the key active components of *P emblica* are all >4.25, indicating binding capability. The binding energies of 2-ethylhexyl ester with MAPK3, luteolin with SRC, kaempferol with MAPK1, fisetin with MAPK1, and quercetin with SRC are above 7.0, indicating extremely strong binding activity. Among them, luteolin, kaempferol, fisetin, chrysin, and quercetin have a binding capability with MAPK3 >9.0. Visualization analysis using Pymol software shows that they can all form stable spatial conformations through hydrogen bonds (Fig. 8).

**Table 3:** Screening of core target proteins.

Core targets	Uniprot ID	PDB ID
SRC	P12931	1A07
AKT1	P31749	1H10
APP	P05067	1BRC
MAPK3	Q16644	3FXW
MAPK1	P28482	7E75

**Table 4:** Binding energies of key active components of *Phyllanthus emblica* with *Phyllanthus emblica*–EP core targets.

Key active components of *Phyllanthus emblica*	Binding energies of *Phyllanthus emblica*–EP core targets (kcal/mol)				
	SRC	AKT1	APP	MAPK3	MAPK1
2-Ethylhexyl ester	−6.3	−5.3	−5.7	−7.1	−5.3
Phyllanthin	−4.7	−4.5	−4.9	−5.7	−6.5
Luteolin	−7.8	−6.3	−6.6	−9.5	−7.7
Kaempferol	−7.5	−6.1	−6.5	−9.6	−7.8
Fisetin	−8.4	−6.5	−6.7	−9.4	−8.1
Chrysin	−7.6	−6.5	−6.8	−9.2	−7.4
Quercetin	−7.6	−6.1	−6.6	−9.6	−7.8

EP = epilepsy.

### 3.6. Ethical approval

The current analysis does not require ethical approval, because our analysis only collects uploaded data information from the public database search.

## 4. Discussion

EP, commonly known as “epileptic seizure” or “epileptic fit.” Traditional Chinese medicine believes that EP is often caused by insufficient innate endowment, emotional imbalance, sudden fright, or head trauma, leading to the dysfunction of the viscera, phlegm obstruction, reversal of Qi, and internal movement of wind and Yang. Modern pharmacological studies have shown that *P emblica* has therapeutic effects on EP. Additionally, modern chemical research has revealed that certain chemical constituents in *P emblica* also contribute to EP treatment,^[27,28]^ and a study^[29]^ indicated that the ellagic acid and gallic acid contained in *P emblica* are extremely strong free radical scavengers. Furthermore, Xie Tao and colleagues^[30]^ found through electrocardiogram recordings that male rats induced with EP by pentylenetetrazole and given epigallocatechin gallate at doses of 25 and 50 mg/kg could effectively inhibit EP seizures. Additionally, Morris water maze experiments showed improved activity in male rats, indicating enhanced cognitive function.

This study utilized network pharmacology methods to identify the main active components of *P emblica* for the treatment of EP, including 2-ethylhexyl ester, phyllanthin, luteolin, kaempferol, fisetin, chrysin, and quercetin. Modern research on the mechanisms of EP treatment has shown that kaempferol has significant anti-inflammatory effects, exerting antioxidant effects by inhibiting MMP9 metalloproteinase, thereby providing neuroprotection.^[31]^ Quercetin alleviates kainic acid-induced EP seizures by inactivating microglia and reducing the production of NF-κB, TNF-α, and IL-1β.^[32]^ Fisetin reduces the grade of EP seizures in rats by providing protection to Na + and K+-ATPase activity.^[33]^ The neuroprotective effect of chrysin nanoparticles against PTZ-induced EP may be due to the alleviation of oxidative stress through the Nrf2/antioxidant response element/HO-1 signaling pathway. Luteolin may increase the threshold for EP seizures by promoting the activation of GABAA receptors and opening gamma-aminobutyric acid (GABA)-mediated chloride channels.^[34]^ Phyllanthin has the potential to counteract EP by stabilizing glutamate excitation and GABA inhibition, modulating brain monoamines, NaK/Ca^+++2^ ATPase, and inhibiting the NF-κB/TLR-4 pathway to improve neuroinflammation (TNF-α, IL-1β, and COX-2).^[35]^ These studies also indicate that the effective components of *P emblica* may improve EP seizures in various aspects, such as antioxidation, regulation of ion channels, and improvement of neuroinflammation. However, there is currently a lack of research on the treatment of EP with 2-ethylhexyl ester, suggesting that *P emblica* may primarily exert its therapeutic effects on EP through phyllanthin, luteolin, kaempferol, fisetin, chrysin, and quercetin.

According to the results of the PPI network and molecular docking, core targets such as SRC, AKT1, APP, MAPK3, and MAPK1 all exhibit good binding energies with the active components of *P emblica*. Relevant research indicates that SRC is abundant in neurons and is associated with proliferation and differentiation during the development of the central nervous system. In vitro EP hippocampal models have shown that the use of SRC pharmacological inhibitors can reduce EP-like discharges.^[36]^ The inhibitory effect of AKT1 on NaV1.1 activity may lead to the occurrence of EP, as it regulates the excitability of the network by controlling the activity of GABAergic interneurons.^[37]^ The reduction of soluble APP has a neuroprotective effect; its absence disrupts calcium homeostasis and increases neuronal excitability, and overexpression of APP can lead to neurodegenerative EP seizures.^[38]^ The MAPK pathway is a modulator of synaptic excitability and has been proven to affect seizures in animal models.^[39]^ In summary, the core targets such as SRC, AKT1, and APP, which were screened in this study, are closely related to EP.

Enrichment analysis and GO analysis results indicate that the treatment of EP with *P emblica* mainly involves the regulation of chemical synaptic transmission, sensory perception of pain, and bicarbonate transport, which are primarily concentrated in aspects such as oxidative stress and inflammation.^[40]^ Studies have reported that inhibiting oxidative damage and neuroinflammation can have a significant neuroprotective effect on PTZ-induced EP.^[28]^ Therefore, the active components of *P emblica* may exert their therapeutic effects on EP by participating in biological processes such as anti-oxidative stress and anti-inflammatory. KEGG analysis results suggest that the pathways related to the treatment of EP with *P emblica* include neuroactive ligand–receptor interaction, nitrogen metabolism, serotonergic synapse, nicotine addiction, retrograde endocannabinoid signaling, and cholinergic synapse. Relevant literature has studied the relationship between these pathways and EP. Disruption of the GABA signaling pathway leads to excessive neuronal excitation and triggers EP seizures, with carbamazepine playing a role by inhibiting the desensitization of AMPA receptors and GABA neurotransmission.^[41]^ Abnormal neurotransmitter–receptor interactions increase susceptibility to EP.^[42]^ GABA transaminase can inhibit EP seizures through cellular nitrogen metabolism.^[43]^ Serotonin blocks EP-like activity in deep entorhinal cortex cells by reducing N-methyl-D-aspartate receptor-mediated excitatory postsynaptic potentials.^[44]^ The nicotine addiction pathway affects neural transmission, immunity, and metabolism, exerting a significant impact on both the central and peripheral nervous systems.^[45]^ The plasticity reorganization of endocannabinoid signaling during the interictal and postictal periods may produce negative neurobiological outcomes related to EP seizure activity.^[46]^ Many genetic EP syndromes are characterized by cholinergic functions that control neuronal excitability and EP threshold.^[47]^ Low concentrations of morphine can inhibit EP seizures, while higher concentrations increase the behavioral activity of spontaneous EP seizures.^[48]^ EP has a bidirectional association with Alzheimer disease (AD), with patients suffering from AD having a 3.1 times higher risk of developing EP, and those with EP having a 1.8 times higher risk of developing AD.^[49]^ Damage at any point in the neurogustatory pathway can lead to taste disorders, with EP being one of the potential causes.^[50]^ WWOX is a tumor suppressor gene that inhibits prostate cancer, and its mutations are a potential mechanism for causing EP seizures, indicating that prostate cancer can affect the occurrence of EP.^[51]^ These studies illustrate that the pathways for treating EP with *P emblica* are related to neuroactive ligand–receptor interaction, nitrogen metabolism, serotonergic synapse, and nicotine addiction, confirming the accuracy of this study.

This study applied network pharmacology methods to predict the active components, targets, and signaling pathways of *P emblica* in treating EP and found that *P emblica* may improve EP seizures through various aspects such as antioxidation, regulation of ion channels, and improvement of neuroinflammation. This lays the foundation for the study of the pharmacological substance basis and mechanism of action of *P emblica* in treating EP and provides a theoretical basis for its clinical application in treating EP.

## Author contributions

**Conceptualization:** Wenjun Chen, Wenlong Guo.

**Data curation:** Wenjun Chen, Hailin Li.

**Formal analysis:** Longfei Xiao, Wenjun Chen.

**Resources:** Longfei Xiao.

**Supervision:** Rong Chen, Qinghua Chen.

**Writing – original draft:** Longfei Xiao.

**Writing – review & editing:** Rong Chen, Qinghua Chen.

## Supplementary Material


