# Supplementary material for: Exploring the mechanism of action of Phyllanthus emblica in the treatment of epilepsy based on network pharmacology and molecular docking
Source: Medicine (Baltimore). 2025 Feb 14;104(7):e41414. doi: 10.1097/MD.0000000000041414 (PMC11835119; doi:10.1097/MD.0000000000041414)
Supplement: Supplementary file 2 [file medi-104-e41414-s002.pdf]

Request for Permission to Publish Content under CC-BY license

Dear Rights Holder or Representative,

I have submitted a paper for publication in ***Medicine*** journal, and wish to include the content listed below in the paper titled *"Exploring the Mechanism of Action of Phyllanthus Emblica in the Treatment of Epilepsy Based on Network Pharmacology and Molecular Docking"*. I'm hereby requesting your permission to include the content in my paper. Please note that all journals are published under a Creative Commons Attribution License (CC BY), which allows for unrestricted use and distribution, even commercial, as long as attribution is given to the creator or rights https://creativecommons.org/licenses/bv/4.0/. holder of the content. See To grant me permission to use the content in my paper, please fill in the information below and then scan the completed form and send it to me at my email address. Thank you.

My name: Longfei Xiao
My email: 1291260129@qq.com

Description of the content which I'm seeking permission to use (citation and/or title, and pasted screen shot, if applicable):

Fig 5 GO Results of Three Ontologies

5A

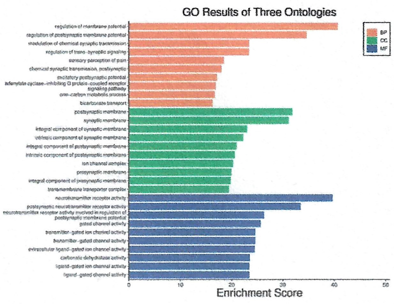

5B

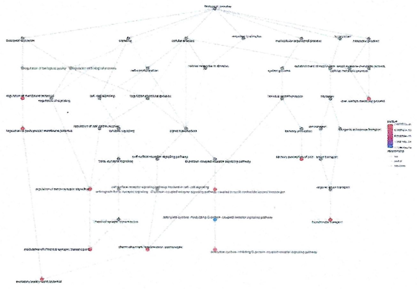

5C

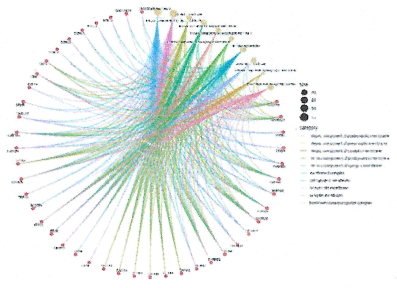

5D

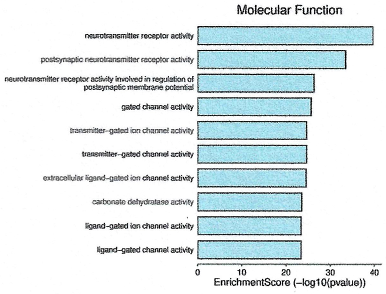

Fig 6 KEGG Enrichment Analysis

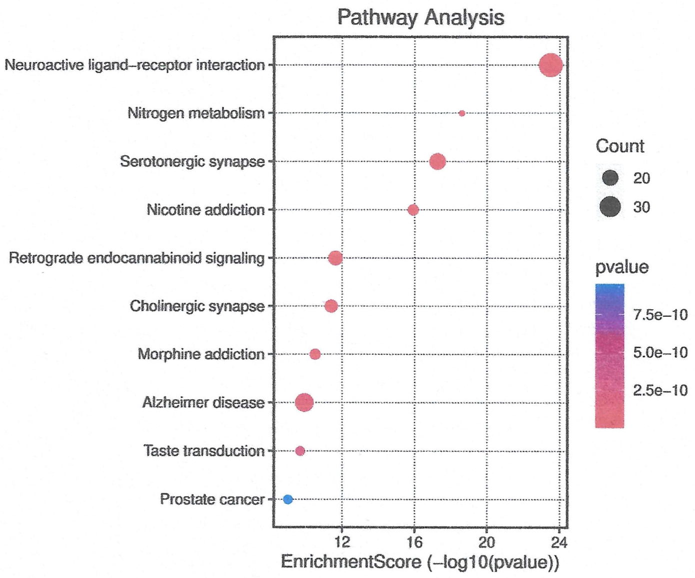

Fig 7 Mapping of Neuroactive Ligand-Receptor Interaction Targets of the hsa04080 Signaling Pathway in Cytokine-Cytokine Receptor Interactions

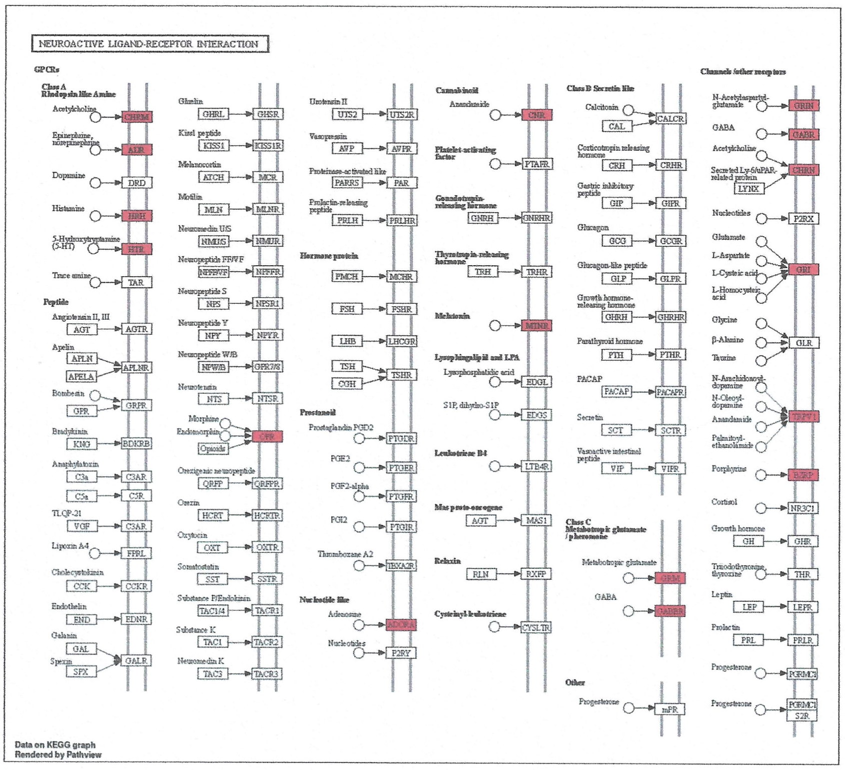

****

On behalf of myself and the rights holder of SRplot (https://www.bioinformatics.com.cn/en), both English and Chinese version. I hereby grant the permission sought herein.

Signature of Party Granting Permission: Mingjie Chen

Date: 2024 11 12

Printed Name and Title: Mingjie Chen
